# Cryptotanshinone Attenuates Inflammatory Response of Microglial Cells via the Nrf2/HO-1 Pathway

**DOI:** 10.3389/fnins.2019.00852

**Published:** 2019-08-21

**Authors:** Yang Zhou, Xiao Wang, Weihai Ying, Danhong Wu, Ping Zhong

**Affiliations:** ^1^Bengbu Medical College, Bengbu, China; ^2^Department of Neurology, Shanghai Traditional Chinese and Western Medicine Hospital, Shanghai University of Traditional Chinese Medicine, Shanghai, China; ^3^Department of Neurology, Shaoxing Central Hospital, Shaoxing, China; ^4^Med-X Research Institute, School of Biomedical Engineering, Shanghai Jiao Tong University, Shanghai, China; ^5^Department of Neurology, Shanghai Fifth People’s Hospital, Fudan University, Shanghai, China

**Keywords:** cryptotanshinone, neuroinflammation, microglial, Nrf2, HO-1, PI3K

## Abstract

Cryptotanshinone (CTN), a monomer compound extracted from the dried roots and rhizomes of *Salvia miltiorrhiza* Bge, has a variety of pharmacological effects. However, little research has been done on the mechanism of CTN in attenuating neuroinflammation. The present study aimed to investigate whether CTN can ameliorate neuroinflammation induced by lipopolysaccharide (LPS) through the Nrf2/heme-oxygenase 1 (HO-1) signaling pathway in BV-2 microglial cells. We found that CTN attenuated the upregulated expression of inducible nitric oxide synthase, cyclooxygenase 2, NOD-like receptor pyrin domains-3, and nitric oxide induced by LPS in microglial cells. In addition, it curtailed the increased release of pro-inflammatory cytokines such as interleukin-1β (IL-1β), IL-6, and tumor necrosis factor-α in LPS-activated microglial cells. Furthermore, CTN significantly increased the levels of NF-κB, Nrf2, HO-1, and Akt proteins. We demonstrated that the anti-inflammatory action of CTN in BV-2 microglial cells was partially through the activation of the Nrf2/HO-1 pathway, which was regulated by the PI3K/Akt signaling pathway. Taken together, our results indicated that CTN downregulated the production and release of proinflammatory mediators in BV-2 microglial cells through activating the Nrf2/HO-1 pathway and subsequently protected neurons from inflammatory injury.

## Introduction

Neuroinflammation and oxidative stress play an important role in the pathogenesis of neurodegenerative diseases such as Alzheimer’s Disease (AD) and Parkinson’s disease (PD). Neuroinflammatory response leads to production of proinflammatory and anti-inflammatory mediators. Regulating the balance between these two types of mediators may avoid or delay the onset of such diseases (Fullerton and Gilroy, 2016; Fülöp et al., 2019). Microglial cells, activated by lipopolysaccharide (LPS), are commonly used for *in vitro* studies on neuroinflammation-related diseases (Velagapudi et al., 2014; Nam et al., 2018). When activated, microglial cells increase the expression of nuclear factor-kappaB (NF-κB) to induce the activity of proinflammatory proteases such as inducible nitric oxide synthase (iNOS) and cyclooxygenase 2 (COX2). As a result, proinflammatory mediators such as nitric oxide (NO), tumor necrosis factor-α (TNF-α), interleukin-1β (IL-1β), and IL-6 were released. Overproduction of these proinflammatory mediators results in neurodegeneration or even neuronal death, accelerating the onset and progression of neurodegenerative diseases (Block et al., 2007). Thus, regulating the activated microglial cells may be a potential therapeutic target for neuroinflammation-related neurodegenerative diseases.

Under the normal condition, multiple anti-oxidative systems counteract oxidative stress and inflammation. NF-E2 p45-related factor 2 (Nrf2, coded by NFE2L2), a member of the human Cap “n” Collar (CNC) basic leucine zipper transcription factor family (Cuadrado et al., 2019) and discovered in 1994, is the key player in defending cells from multiple stress-related injuries. Under the physiological condition, Nrf-2 binds to Kelch-like ECH-associated protein 1 (Keap1) and is degraded continuously. However, under stress conditions, it dissociates from Keap-1 and translocates to the nucleus (Loboda et al., 2016), increasing the gene expression of anti-inflammatory mediators. Apart from this, Nrf-2 induces the expression of heme-oxygenase 1 (HO-1) (Na and Surh, 2014). HO-1, a type of heat shock protein, has the anti-oxidative and anti-inflammatory potential. It has been proven that increasing the activity of HO-1 can ameliorate inflammatory response and inhibiting its activity leads to aggravated inflammatory injury (Wu et al., 2014; Yin et al., 2015). Therefore, the Nrf-2/HO-1 signaling pathway is an important therapeutic target for effectively managing neuroinflammation-related neurodegenerative diseases.

Cryptotanshinone (CTN), a monomer compound extracted from the dried roots and rhizomes of *Salvia miltiorrhiza* Bge, exhibits anti-oxidative (Wang W. et al., 2018) and anti-inflammatory effects (Feng et al., 2017). It inhibits the activation of NF-κB induced by LPS in macrophages (Tang et al., 2011) as well as the activity of COX2. In addition, it decreases the expression of endothelin-1, leading to ameliorated inflammatory response (Jin et al., 2006). In Caco-2 cells, CTN exerts anti-inflammatory effects through the toll-like receptor 4 (TLR4)/NF-κB pathway (Cao et al., 2018). It has been shown that CTN attenuates the increased production of NO induced by LPS in BV-2 microglial cells (Lee et al., 2006). Little research has been done on the underlying mechanisms other than this. Therefore, the present study aimed to investigate whether CTN can inhibit inflammatory response of BV-2 microglial cells induced by LPS through the Nrf-2/HO-1 signaling pathway.

## Materials and Methods

### Materials

Cryptotanshinone (C5624) and LPS (L2880) were purchased from Sigma–Aldrich (St. Louis, MO, United States). LY294002 (HY-10108) was purchased from MedChemExpress (Shanghai, China). LY294002 is a PI3K/Akt pathway inhibitor CTN and LY294002 were dissolved in dimethyl sulfoxide to a stock concentration of 10 mM/l and LPS was dissolved in the phosphate buffer saline (PBS) to a stock concentration of 1 mg/ml. All of them were frozen in a −20°C freezer before use. Nrf2 siRNA, HO-1 siRNA, and the control siRNA were purchased from GenePharma (Shanghai, China).

### Cells Culture

Murine BV-2 microglial cells were obtained from Institute of Neurology, Ruijin Hospital (Shanghai, China) and were cultured in Dulbecco’s Modified Eagle Medium (DMEM) (HyClone, Logan, UT, United States) containing 10% fetal bovine serum (FBS; Gibco, Carlsbad, CA, United States). 100 U/ml penicillin and 100 μg/ml streptomycin were added into the culture medium in order to prevent bacterial infection. Cells were maintained in a 37°C incubator supplied with 5% CO_2_ and 95% air.

### Intracellular Lactate Dehydrogenase (LDH) Assay

The intracellular LDH assay was conducted as previously described (Ma et al., 2014) in order to test the viability of cells. Briefly, cells were lysed in a lysis buffer containing 0.04% Triton X-100, 2 mM HEPES, 0.01% bovine serum albumin (pH 7.5) for 20 min after washing with PBS. 40 μl cell lysate was mixed with 200 μl potassium phosphate buffer (500 mM, pH 7.5) containing 0.3 mM NADH and 2.5 mM sodium pyruvate before reading the absorbance at 340 nm on a microplate photometer.

### Extracellular LDH Assay

The extracellular LDH assay was conducted as previously described (Ma et al., 2014) to determine the level of cell death. Briefly, 50 μl cell culture supernatant was mixed with 160 μl potassium phosphate buffer (500 mM, pH 7.5) containing 0.3 mM β-Nicotinamide adenine dinucleotide, reduced dipotassium salt (NADH) and 2.5 mM sodium pyruvate, followed by reading the absorbance at 340 nm using a microplate photometer.

### Morphological Analysis of BV-2 Microglial Cells

BV-2 microglial cells were pretreated with CTN for 1 h, followed by being stimulated with LPS for 18 h. The control group was treated with the phosphate buffer for 19 h. Then the cell morphology was observed using a light microscope.

### Nitric Oxide Assay

The level of NO was determined using a NO Test Kit (Beyotime, Jiangsu, China) by following the manufacturer’s instructions. Equal volumes of cell culture medium, Griess reagent I, and Griess reagent II were mixed before reading the absorbance at 540 nm using a microplate photometer. Concentrations of nitrite in the samples were calculated by normalizing them to concentrations of the standards and concentrations of proteins were tested using the bicinchoninic acid (BCA) assay.

### Real-Time PCR Assay

Total RNA of BV-2 microglial cells was extracted with a total RNA extraction kit (Bioteck Pharma, Beijing, China) by following the manufacturer’s instructions after washing cells with PBS. Concentrations and purity of total RNA were measured using a spectrophotometer at 260 and 280 nm. One microgram of total RNA was reverse transcribed into cDNA using a Prime-Script RT kit (Takara Bio, Dalian, China). cDNA was then mixed with reagents of a SYBR Premix Ex Taq kit (Takara Bio, Dalian, China) before running real-time PCR on an ABI 7900HT machine. The sequences of primers were:

IL-1β (sense 5′-AAGGGCTGCTTCCAAACCTTTGAC-3′,

anti-sense 5′-ATACTGCCTGCCTGAAGCTCTTGT-3′);

IL-6 (sense 5′-TCCATCCAGTTGCCTTCTTG-3′,

anti-sense 5′-AAGCCTCCGACTTGTGAAGTG-3′);

TNF-α (sense 5′-CCCTCACACTCAGATCATCTTCT-3′,

anti-sense 5′-GCTACGACGTGGGCTACAG-3′);

GAPDH (sense 5′-CCTGCACCACCAACTGCTTA-3′,

anti-sense 5′-GGCCATCCACAGTCTTCTGA-3′).

Data were analyzed by comparing the threshold cycles (Ct) of target genes which were normalized to their corresponding Ct of GAPDH.

### Western Blot

Cells were washed with PBS and lysed in the radio immunoprecipitation assay (RIPA) buffer (Millipore, Temecula, CA, United States) containing 1% Complete Protease Inhibitor (CWBIO, Shanghai, China) and 1 mM phenylmethanesulfonyl fluoride (PMSF). The lysates were centrifuged at 15,000 rpm for 10 min at 4°C before testing their concentrations of proteins using the BCA assay kit (Thermo Scientific, Waltham, MA, United States). Thirty micrograms of total protein was separated using SDS–PAGE (10% gel) and the proteins were transferred to a nitrocellulose membrane (0.45 μm, Millipore, Temecula, CA, United States). After blocking with 5% skimmed milk for 1 h, the membrane was sequentially incubated in the primary and the corresponding HRP conjugated secondary antibody solutions (1:4000, Epitomics, Zhejiang, China) for overnight at 4°C and 1 h at room temperature, respectively. Finally, target proteins were visualized using the ECL detection reagents and photographs were taken. The primary antibodies were: Akt (1:1000, Cell Signaling Technology, Danvers, MA, United States), phospho-Akt (1:1000, Cell Signaling Technology, Danvers, MA, United States), Nrf2 (1:1000, Cell Signaling Technology, Danvers, MA, United States), NOD-like receptor pyrin domains-3 (NLRP3) (1:1000, Cell Signaling Technology, Danvers, MA, United States), iNOS (1:1000, Abcam), COX2 (1:1000, Abcam), tubulin (1:2000, Abcam), Lamin A/C (1:5000, Abcam), NF-κB p65 (1:20,000, Abcam), and HO-1(1:5000, Abcam).

### Cytoplasmic and Nuclear Protein Extraction

Cells were washed with PBS and centrifuged at 15,000 rpm for 10 min at 4°C. Nuclear and cytoplasmic proteins were extracted separately using a commercial kit (Beyotime, Jiangsu, China) by following the manufacturer’s instructions. Concentrations of cytoplasmic and nuclear proteins were quantified using the BCA assay kit.

### Immunofluorescence Assay

BV-2 microglial cells were seeded onto sterile glass coverslips in 24-well culture plates. After attaching to the coverslips, cells were pretreated with CTN (5 and 10 μM) for 1 h prior to incubation with LPS (1 μg/ml) for 30 min, 1 h, or 18 h. Cells were fixed with 4% paraformaldehyde for 20 min and blocked with 10% normal goat serum in PBS for 30 min at room temperature after being permeabilized with 0.2% Triton X-100 for 20 min at room temperature. Cells were sequentially incubated in the primary antibody (NF-κB 1:100, Nrf2 1:200, HO-1 1:250, overnight at 4°C) and the appropriate fluorophore-conjugated secondary antibody solutions (Alexa Fluor 488 conjugated, 1:500, Invitrogen, Carlsbad, CA, United States, 1 h at room temperature) before being counterstained with DAPI (1:300, Beyotime, Jiangsu, China). Finally, the cells were washed with PBS and coverslipped with a fluorescent mounting medium containing the anti-fading reagent. Images were then captured using a confocal laser scanning microscope (Leica TCS SP5 II, Germany).

### Nrf2 and HO-1 siRNA Transient Transfections

BV-2 microglial cells were cultured in 24-well culture plates. When cell confluence reached 40–60%, cells were transfected with Nrf2 siRNA, HO-1 siRNA, and control siRNA, respectively, using the siRNA transfection reagent Lipofectamine 3000 (Invitrogen, Carlsbad, CA, United States) by following the manufacturer’s instructions. Six hours later, the transfected cells were stimulated with LPS as described above after pretreatment with or without CTN for 1 h, respectively.

### Statistical Analyses

All experiments were repeated at least three times. Data were expressed as mean ± SEM. Data were analyzed using one-way ANOVA followed by Student–Newman–Keuls *post hoc* test. It was considered statistically significant when *p*-value was <0.05.

## Results

### Effect of CTN on the Viability of BV-2 Microglial Cells

In the present study, different concentrations of CTN (Supplementary Figure S1A) were tested using the intracellular or extracellular LDH assay in order to find the optimal concentration of CTN. BV-2 microglial cells were pretreated with CTN for 1 h, followed by being stimulated with LPS for 24 h with CTN being present or absent. It was found that CTN in the range of 1–10 μM was innocuous to BV-2 microglial cells, but toxic at the concentration of 20 μM or over (Supplementary Figure S1B). Therefore, 5 and 10 μM were used in the following experiments.

### Effect of CTN on the Morphology of LPS-Activated BV-2 Microglial Cells

Morphological analysis was performed to assess changes of BV-2 microglial cells before and after drug treatment. Microglial cells in the normal condition were round in shape with few branches, whereas microglial cells stimulated with LPS had significantly larger and longer cell body areas with longer perimeters. When BV2 microglial cells were pretreated with CTN for 1 h and then treated with LPS for 18 h, the perimeter of BV2 microglia was significantly shortened compared with that of microglial cells stimulated with LPS, but the area did not significantly decrease (Supplementary Figure S2).

### Influence of CTN on LPS-Induced NO Production by BV-2 Microglial Cells

**FIGURE 1:**
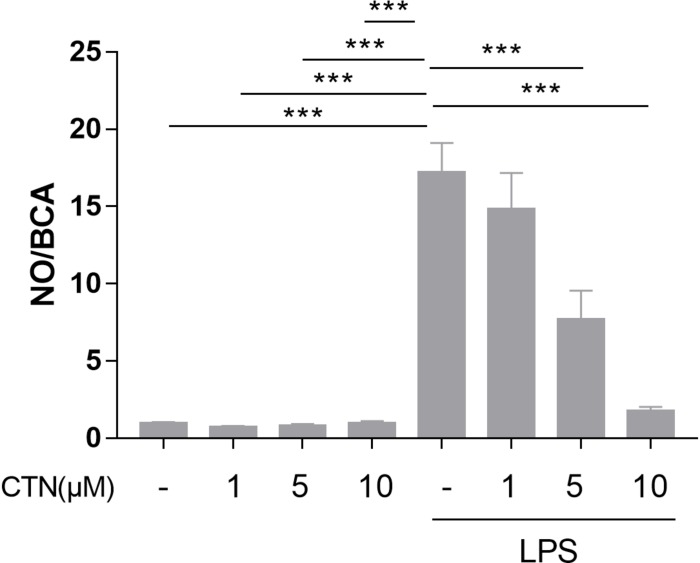
Effect of CTN on LPS-induced NO production in BV-2 microglial cells. BV-2 microglial cells were pretreated with CTN at various concentrations for 1 h and subsequently co-treated with 1 μg/ml LPS for 24 h. CTN at concentrations of 1, 5, and 10 μM did not influence the amount of NO produced by BV-2 microglial cells. LPS significantly increased the amount of NO, which was significantly attenuated by CTN in a dose-dependent manner. Data were presented as mean ± SEM. All experiments were repeated at least three times. ^∗∗∗^*P* < 0.001.

To determine the influence of CTN on LPS-induced NO production, BV2 microglial cells were pretreated with CTN for 1 h and then co-treated with LPS for 24 h. It was found that LPS significantly increased the level of NO, which was attenuated by CTN in a dose-dependent manner (Figure 1).

### Effect of CTN on LPS-Induced Expression of iNOS and COX2 in BV-2 Microglial Cells

**FIGURE 2:**
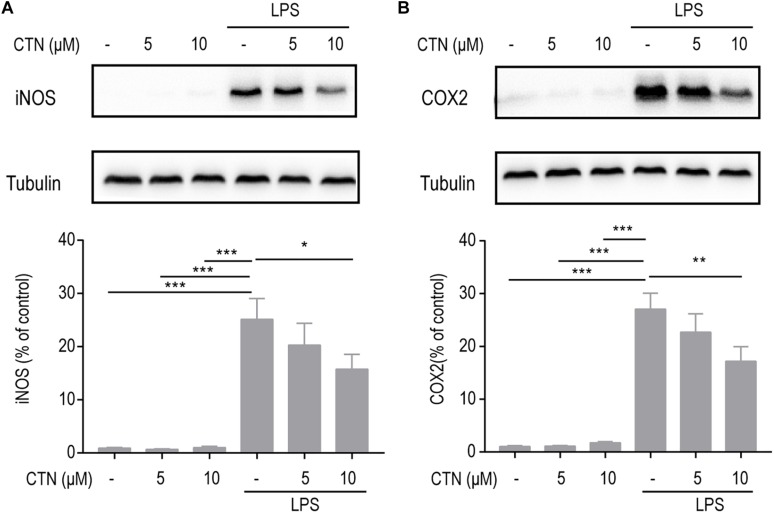
Effect of CTN on LPS-induced expression of iNOS and COX2 in BV2 microglial cells. BV-2 microglial cells were pretreated with CTN at various concentrations for 1 h and subsequently co-treated with 1 μg/ml LPS for 18 h. CTN at concentrations of 5 and 10 μM did not influence the expression of iNOS **(A)** or COX2 **(B)** produced by BV-2 microglial cells. LPS significantly increased the levels of iNOS and COX2, which were significantly attenuated by CTN in a dose-dependent manner. Data were presented as mean ± SEM. All experiments were repeated at least three times. ^∗^*P* < 0.05, ^∗∗^*P* < 0.01, ^∗∗∗^*P* < 0.001.

Inducible nitric oxide synthase and COX2 are two pro-inflammatory proteases produced by activated microglial cells and they play an important role in neuroinflammation as inflammatory mediators. To examine the effect of CTN on LPS-induced expression of iNOS and COX2, BV-2 microglial cells were pretreated with CTN (5 and 10 μM) for 1 h and then co-treated with LPS for 18 h. Western blot results showed that LPS significantly increased the levels of iNOS (Figure 2A) and COX2 (Figure 2B) in BV-2 microglial cells, which were significantly downregulated by CTN in a dose-dependent manner.

### Effect of CTN on LPS-Induced Expression of IL-1β, IL-6, and TNF-α in BV-2 Microglial Cells

**FIGURE 3:**
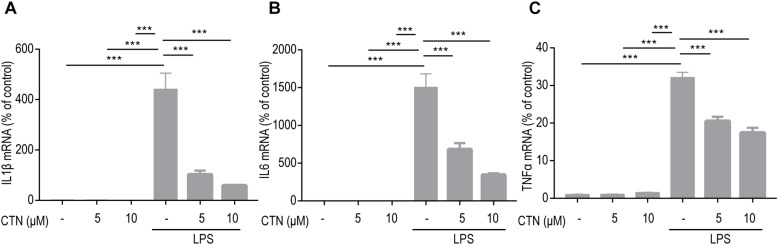
Effect of CTN on LPS-induced expression of IL-1β, IL-6, and TNF-α in BV2 microglial cells. BV-2 microglial cells were pretreated with CTN at various concentrations for 1 h and subsequently co-treated with 1 μg/ml LPS for 6 h. Five or 10 μM CTN alone did not influence the expression of IL-1β **(A)**, IL-6 **(B)**, and TNF-α **(C)** in BV-2 microglial cells. LPS significantly increased the expression of these three genes, which was significantly attenuated by 5 or 10 μM CTN. Data were presented as mean ± SEM. All experiments were repeated at least three times. ^∗∗∗^*P* < 0.001.

Cytokines such as IL-1β, IL-6, and TNF-α play an important role in inflammation. To investigate whether CTN can prevent or delay the onset of neurodegenerative diseases as well as their progression by decreasing the production and release of inflammatory cytokines, RT-PCR was performed to test the levels of genes of inflammatory cytokines. It was observed that levels of IL-1β, IL-6, and TNF-α mRNA were significantly increased by LPS. CTN significantly downregulated levels of IL-1β (Figure 3A), IL-6 (Figure 3B), and TNF-α (Figure 3C) in BV-2 microglial cells induced by LPS in a dose-dependent manner.

### Effects of CTN on LPS-Induced Activation of NF-κB in BV-2 Microglial Cells

**FIGURE 4:**
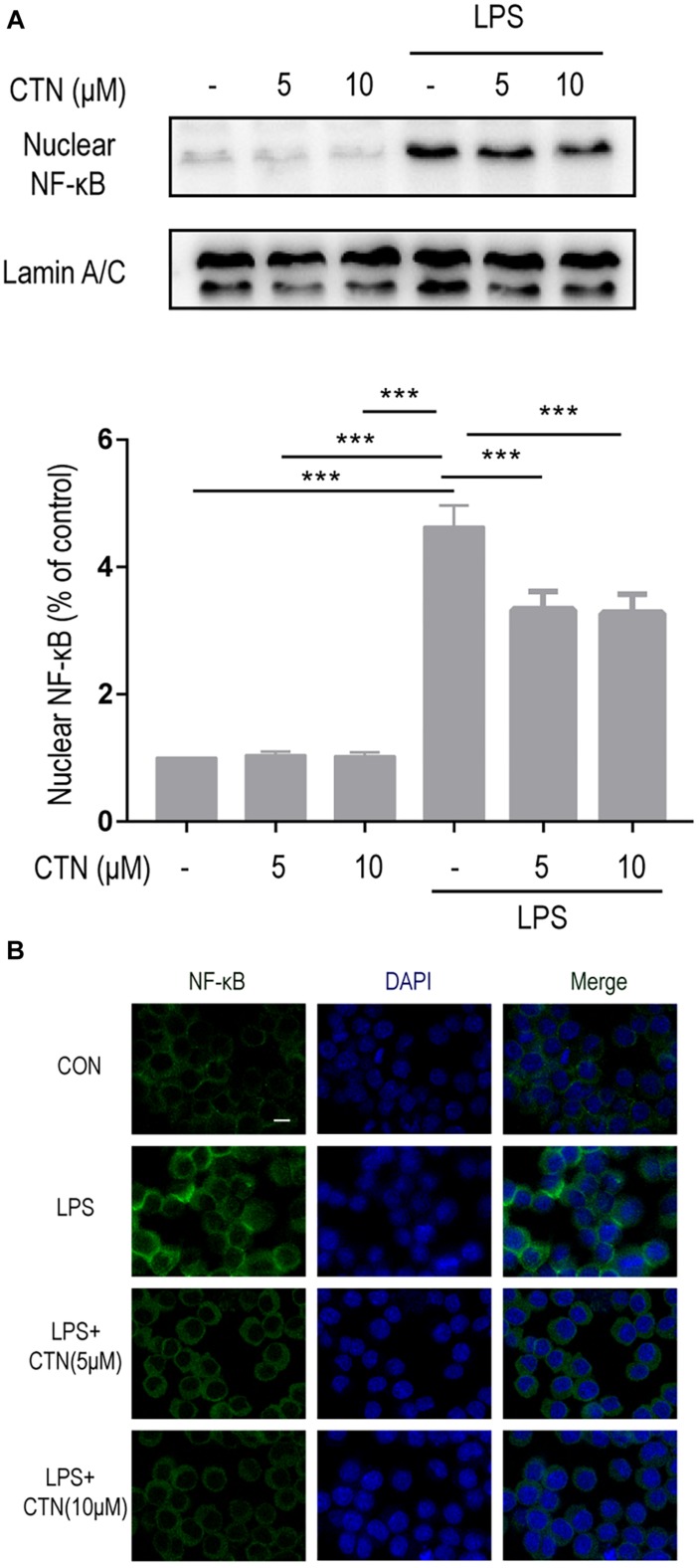
Effect of CTN on LPS-induced activation of NF-κB in BV2 microglial cells. BV-2 microglial cells were pretreated with CTN at various concentrations for 1 h and subsequently co-treated with 1 μg/ml LPS for 30 min. **(A)** Five or 10 μM CTN alone did not influence the expression of NF-κB in BV-2 microglial cells. LPS significantly increased the expression of NF-κB, which was significantly attenuated by 5 or 10 μM CTN. Data were presented as mean ± SEM. All experiments were repeated at least three times. ^∗∗∗^*P* < 0.001. **(B)** Immunofluorescence staining against NF-κB showing similar results to those in **(A)**. Green is NF-κB staining, blue is DAPI counterstain (magnification, 40×, scale bar: 20 μm). All experiments were repeated at least three times.

It is well known that induction of many proinflammatory cytokines is primarily relying on the activation of NF-κB. In the present study, the level of NF-κB in BV-2 microglial cells was tested after pretreating these cells with CTN for 1 h and then co-treating with LPS for 30 min. Western blot showed that LPS significantly increased the level of NF-κB, indicating that LPS induced the activation of NF-κB. CTN significantly downregulated the level of NF-κB, suggesting an inhibitory effect on the activity of NF-κB (Figure 4A). Immunofluorescence staining further confirmed the result from Western blot (Figure 4B).

### Effect of CTN on LPS-Induced Expression of Nrf2 and HO-1 in BV-2 Microglial Cells

**FIGURE 5:**
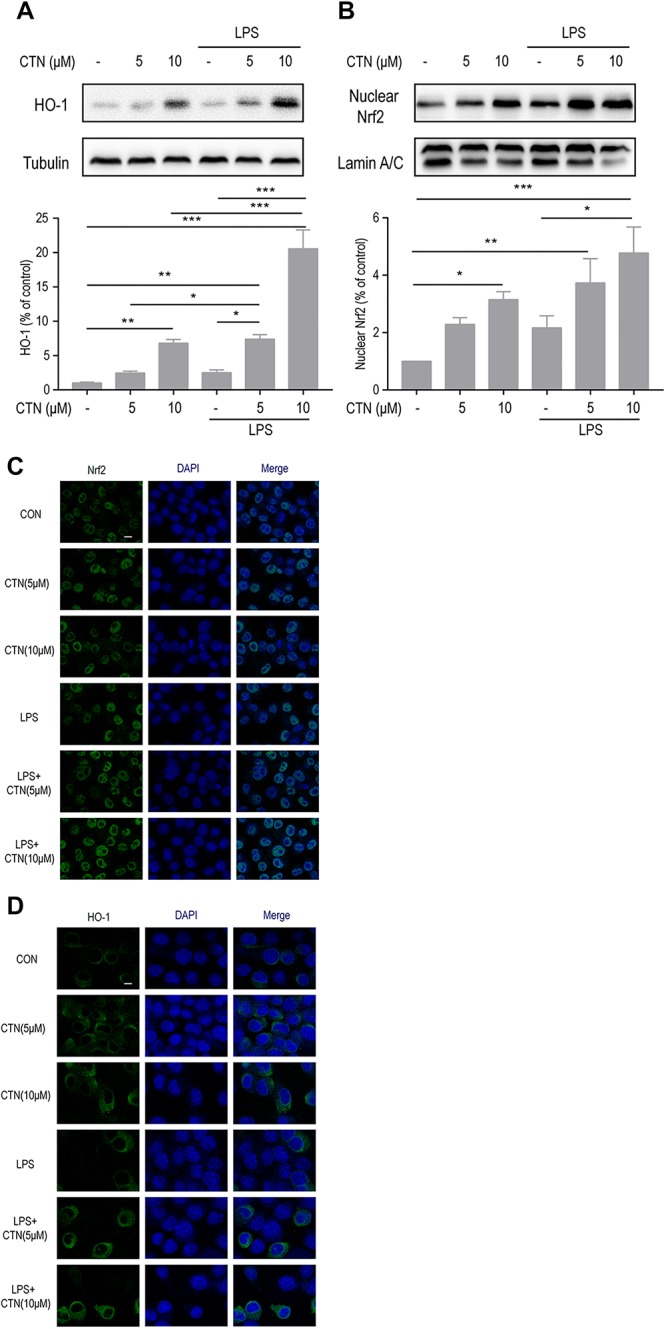
Effect of CTN on LPS-induced expression of Nrf2 and HO-1 in BV2 microglial cells. **(A)** BV-2 microglial cells were pretreated with CTN at various concentrations for 1 h and subsequently co-treated with 1 μg/ml LPS for 18 h. Ten micromolars of CTN significantly increased the expression of HO-1 in BV-2 microglial cells. LPS did not significantly increased the expression of HO-1, but augmented the increase induced by 5 or 10 μM CTN. Data were presented as mean ± SEM. All experiments were repeated at least three times. ^∗^*P* < 0.05, ^∗∗^*P* < 0.01, ^∗∗∗^*P* < 0.001. **(B)** BV-2 microglial cells were pretreated with CTN at various concentrations for 1 h and subsequently co-treated with 1 μg/ml LPS for 1 h. Five micromolars of CTN alone did not influence the expression of Nrf2 in BV-2 microglial cells, but 10 μM CTN significantly increased the expression of Nrf2. LPS did not significantly increase the expression of Nrf2, but augmented the increase induced by 5 or 10 μM CTN. **(C)** Immunofluorescence staining against Nrf2 showing similar results to those in **(B)**. Green is Nrf2 staining, blue is DAPI counterstain (magnification, 40×, scale bar: 20 μm). All experiments were repeated at least three times. **(D)** Immunofluorescence staining against HO-1 showing similar results to those in **(A)**. Green is HO-1 staining, blue is DAPI counterstain (magnification, 40×, scale bar: 20 μm). All experiments were repeated at least three times.

To investigate the underlying anti-inflammatory mechanism of CTN, we further examined the effect of CTN on LPS-induced levels of Nrf2 and HO-1 proteins. Western blot showed that CTN significantly increased the levels of Nrf2 (Figure 5B) and HO-1 in a dose-dependent manner (Figure 5A), LPS did not significantly increase the expression of Nrf2 and HO-1, but augmented the increase induced by 5 or 10 μM CTN. This was confirmed by immunofluorescence analysis (Figures 5C,D).

### The Anti-inflammatory Effect of CTN Is Dependent on Nrf2

**FIGURE 6:**
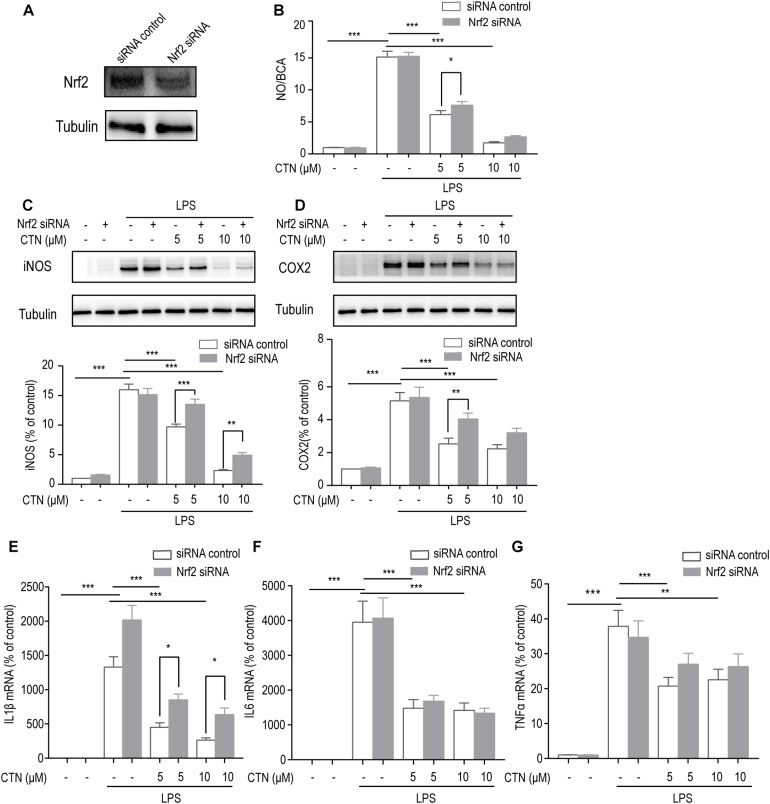
The anti-inflammatory effect of CTN is partially through Nrf2. **(A)** Expression of Nrf2 was significantly decreased after transfecting cells with Nrf2 siRNA. **(B)** BV2 microglial cells were treated with CTN at various concentrations for 1 h in the presence or absence of Nrf2 siRNA and subsequently co-treated with 1 μg/ml LPS for 24 h. LPS significantly increased the amount of NO in the presence or absence of Nrf2 siRNA, which was significantly attenuated by CTN at 5 μM. Transfection with Nrf2 siRNA partially reversed CTN’s inhibitory effect. **(C,D)** BV2 microglia cells were treated with CTN at various concentrations for 1 h in the presence or absence of Nrf2 siRNA and subsequently co-treated with 1 μg/ml LPS for 18 h. LPS significantly increased the expression of iNOS **(C)** and COX2 **(D)** in the presence or absence of Nrf2 siRNA, which was significantly attenuated by CTN. Transfection with Nrf2 siRNA partially reversed CTN’s inhibitory effect. **(E–G)** BV2 microglia cells were treated with CTN at various concentrations for 1 h in the presence or absence of Nrf2 siRNA and subsequently co-treated with 1 μg/ml LPS for 6 h. LPS significantly increased the expression of IL-1β **(E)**, IL-6 **(F)**, and TNF-α **(G)** in the presence or absence of Nrf2 siRNA, which was significantly attenuated by CTN at 5 or 10 μM. Transfection with Nrf2 siRNA partially reversed CTN’s inhibitory effect on the expression of IL-1β. Data were presented as mean ± SEM. All experiments were repeated at least three times. ^∗^*P* < 0.05, ^∗∗^*P* < 0.01, and ^∗∗∗^*P* < 0.001.

To determine whether the anti-inflammatory effect of CTN is dependent on the activation of Nrf2, the level of Nrf2 was decreased by knocking down Nrf2 using Nrf2 siRNA. Western blot showed that Nrf2 siRNA significantly inhibited the expression of Nrf2 (Figure 6A). The NO assay showed that Nrf2 siRNA significantly reversed the inhibitory effect of CTN on NO production (Figure 6B). Similarly, the level of IL-1β mRNA (Figure 6E) and levels of iNOS (Figure 6C) and COX2 (Figure 6D) were reversed by Nrf2 siRNA. However, levels of IL6 (Figure 6F) and TNF-α mRNA (Figure 6G) were not significantly reversed by Nrf2 siRNA. Taken together, the anti-inflammatory effect of CTN was partially dependent on the activation of Nrf2.

### The Anti-inflammatory Effect of CTN Is Dependent on HO-1

**FIGURE 7:**
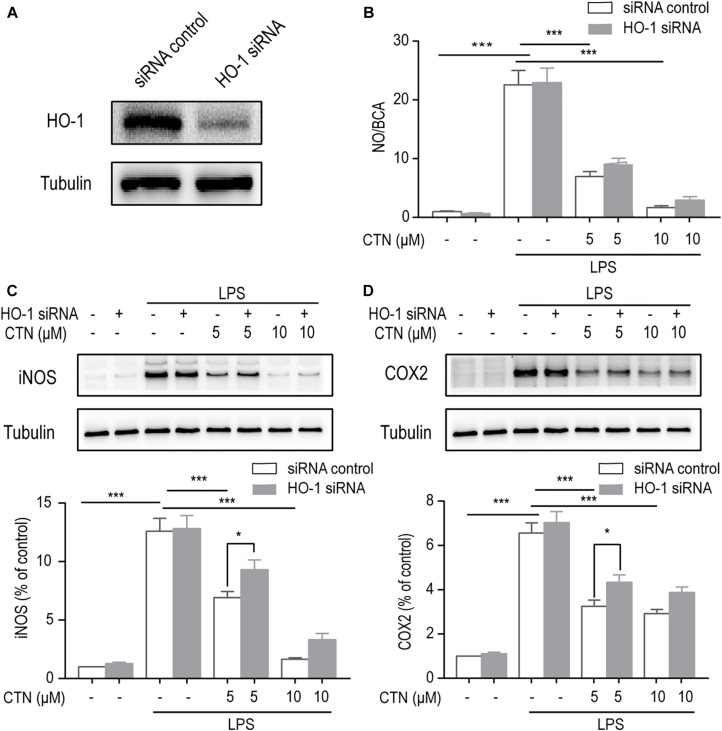
The anti-inflammatory effect of CTN is partially through HO-1. **(A)** Expression of HO-1 was significantly decreased after transfecting cells with HO-1 siRNA. **(B)** BV2 microglial cells were treated with CTN at various concentrations for 1 h in the presence or absence of HO-1 siRNA and subsequently co-treated with 1 μg/ml LPS for 24 h. LPS significantly increased the level of NO in the presence or absence of HO-1 siRNA, which was significantly attenuated by CTN at 5 or 10 μM. Transfection with HO-1 siRNA did not significantly reverse CTN’s inhibitory effect. **(C,D)** BV2 microglia cells were treated with CTN at various concentrations for 1 h in the presence or absence of HO-1 siRNA and subsequently co-treated with 1 μg/ml LPS for 18 h. LPS significantly increased the expression of iNOS **(C)** and COX2 **(D)** in the presence or absence of HO-1 siRNA, which was significantly attenuated by CTN at 5 and 10 μM. Transfection with HO-1 siRNA partially reversed CTN’s inhibitory effect. Data were presented as mean ± SEM. All experiments were repeated at least three times. ^∗^*P* < 0.05, ^∗∗∗^*P* < 0.001.

To determine whether the anti-inflammatory effect of CTN is dependent on the activation of HO-1, the level of HO-1 was decreased by knocking down HO-1 using HO-1 siRNA. Western blot showed that HO-1 siRNA significantly inhibited the expression of HO-1 (Figure 7A). The NO assay showed that HO-1 siRNA partially reversed the inhibitory effect of CTN on NO production, but this effect was non-significant (Figure 7B). Western blot showed the reversal of iNOS (Figure 7C) and COX2 (Figure 7D) expression downregulated by CTN. Taken together, the anti-inflammatory effect of CTN was partially dependent on HO-1.

### CTN-Induced Upregulation of Nrf2 and HO-1 Depends on the PI3K/Akt Pathway

**FIGURE 8:**
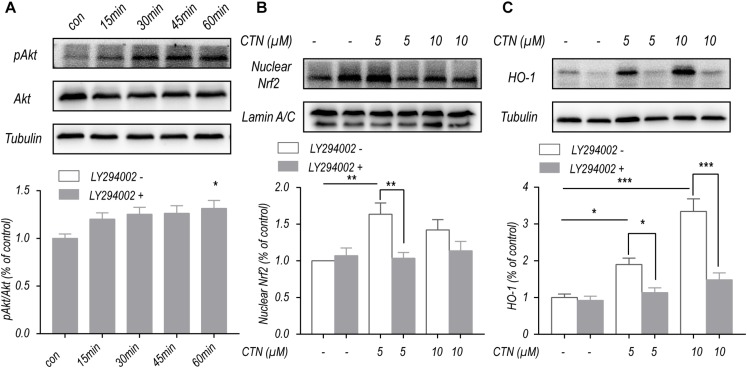
CTN increases the expression of Nrf2 and HO-1 partially through the PI3K/Akt signaling pathway. **(A)** Five micromolars of CTN increased the level of pAkt/Akt with time. **(B)** BV2 microglial cells were treated with CTN at various concentrations for 1 h in the presence or absence of LY294002 (20 μM) for 3 h and subsequently co-treated with 1 μg/ml LPS for 1 h. Five micromolars of CTN increased the level of nuclear Nrf2 in BV-2 microglial cells, which was significantly attenuated by LY294002 in the 5 μM group, but not in the 10 μM group. **(C)** BV2 microglia cells were treated with CTN at various concentrations for 1 h in the presence or absence of LY294002 (20 μM) for 3 h and subsequently co-treated with 1 μg/ml LPS for 18 h. Five or 10 μM CTN significantly increased the expression of HO-1 in BV-2 microglial cells, which was significantly attenuated by LY294002. Data were presented as mean ± SEM. All experiments were repeated at least three times.^∗^*P* < 0.05, ^∗∗^*P* < 0.01, ^∗∗∗^*P* < 0.001.

To investigate the effect of CTN on Akt phosphorylation, BV-2 microglial cells were treated with CTN at multiple time points. Western blot showed that CTN increased the phosphorylation of Akt with time (Figure 8A). To determine whether upregulation of Nrf2 and HO-1 by CTN is dependent on the PI3K/Akt pathway, LY294002, a PI3K/Akt pathway inhibitor, was added to the culture medium. It was found that LY294002 downregulated CTN-induced expression of Nrf2 (Figure 8B) and HO-1 (Figure 8C). Therefore, CTN-induced expression of Nrf2 and HO-1 was partially dependent on the activation of the PI3K/Akt pathway.

### The Inhibitory Effect of CTN on NLRP3 Inflammasomes Is Dependent on the Activation of Nrf2

**FIGURE 9:**
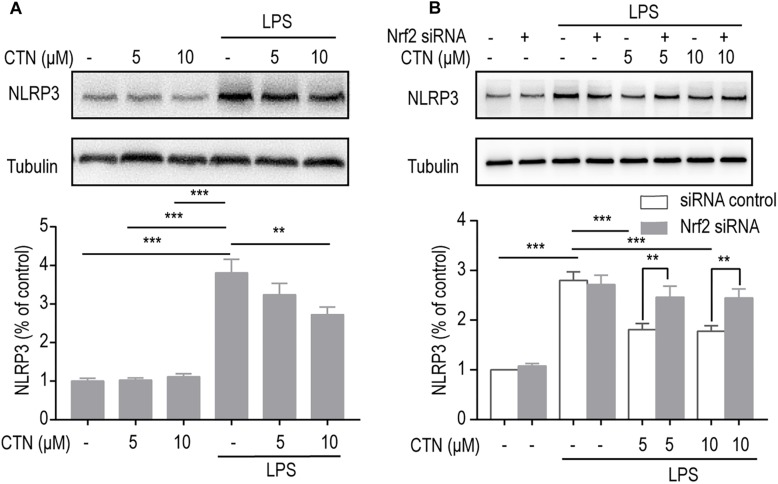
The inhibitory effect of CTN on NLRP3 inflammasomes is partially through Nrf2. **(A)** BV2 microglia cells were treated with CTN at various concentrations for 1 h and subsequently co-treated with 1 μg/ml LPS for 18 h. CTN at concentrations of 5 or 10 μM did not influence the expression of NLRP3, whereas LPS significantly increased the expression of NLRP3, which was attenuated by 10 μM CTN, but not by 5 μM CTN. **(B)** BV2 microglia cells were treated with CTN at various concentrations for 1 h in the presence or absence of Nrf2 siRNA and subsequently co-treated with 1 μg/ml LPS for 18 h. After transfecting cells with Nrf2 siRNA, the inhibitory effect of 5 μM CTN was partially reversed. Data were presented as mean ± SEM. All experiments were repeated at least three times. ^∗∗^*P* < 0.01, ^∗∗∗^*P* < 0.001.

NOD-like receptor pyrin domains-3 inflammasomes are important players in the development of inflammation. To investigate the effect of CTN on LPS-induced expression of NLRP3 in BV-2 microglial cells, Western blot was performed after pretreating BV-2 microglial cells with CTN (5 and 10 μM) for 1 h and then co-treating with LPS for 18 h. It was shown that LPS significantly increased the level of NLRP3 protein, which was attenuated by CTN (Figure 9A). When the level of Nrf2 was downregulated by Nrf2 siRNA, the inhibitory effect of CTN on NLRP3 was partially reversed (Figure 9B).

## Discussion

Cryptotanshinone is an important component of the extract from the dry root and rhizomes of *S. miltiorrhiza* Bge. It is a small lipophilic molecule that easily passes the blood–brain barrier. It has shown multiple pharmacological effects such as anti-inflammatory (Feng et al., 2017) and anti-oxidative properties (Wang W. et al., 2018). However, its potential in anti-inflammatory response has not been comprehensively studied. The present study found that CTN effectively ameliorated LPS-induced inflammatory response of BV-2 microglial cells. We are the first to reveal the signaling pathways involved in its anti-inflammatory effect. We found that CTN downregulated LPS-induced expression of proinflammatory mediators such as iNOS and COX-2, as well as the production and release of NO, IL-1β, IL-6, and TNF-α. Apart from these, we found that CTN inhibited the NLRP3 inflammasome and its anti-inflammatory effect was related to the expression and activation of NF-κB, Nrf2, and HO-1. The other key finding is that CTN-induced upregulation of Nrf2 and HO-1 was dependent on the activation of the PI3K/Akt signaling pathway.

Microglial cells are the key inflammatory cells in the brain. Under the normal condition, microglial cells are in a resting state with apparent ramifications. When stimulated, these cells are converted to an amoeboid shape with few ramifications and larger cell bodies, indicating an activated state. Endotoxins-activated microglial cells can release diverse proinflammatory mediators, which contribute to the onset and progression of neurodegenerative diseases (Lull and Block, 2010). LPS-activated microglial cells have been widely used as an *in vitro* model of neuroinflammatory diseases due to their release of multiple inflammatory mediators (Velagapudi et al., 2014; Nam et al., 2018). In the present study, we first observed that CTN suppressed LPS-induced morphological changes in BV2 microglial cells, indicating that CTN inhibited microglial activation induced by LPS. We also demonstrated that CTN inhibited the upregulation of NO, TNF-α, IL-1β, and IL-6, which might attribute to the inhibited expression of their enzymes such as iNOS and COX2 in BV-2 microglial cells.

NF-κB is widely expressed in the central nervous system and is a key regulator of inflammatory and immune responses as well as expression of multiple genes such as iNOS and COX2. It also promotes the secretion of a variety of proinflammatory cytokines, such as TNF-α and IL-1β (Yamamoto and Gaynor, 2001; Li and Verma, 2002). Under the physiological condition, NF-κB binds to its inhibitor I-κB and stays dormant in the cytoplasm. Once activated, it dissociates from I-κB and translocates to the nucleus where it initiates the transcription of downstream genes. It is our finding that LPS increased the activity of NF-κB and subsequently increased the expression of iNOS and COX2. CTN downregulated the increased expression of these two proinflammatory mediators by inhibiting the activity of NF-κB. As a result, the secretion of proinflammatory mediators was decreased.

We are the first to demonstrate that the anti-inflammatory effect of CTN was partially through the activation of Nrf2/HO-1. It is known that Nrf2-ARE is one of the protecting mechanisms of neurodegenerative diseases by attenuating neuroinflammation (Buendia et al., 2016; Draheim et al., 2016). It has been shown that multiple drugs decrease the secretion of proinflammatory mediators by increasing the level of Nrf2 and thereby ameliorate neuroinflammation (Wang X. et al., 2018; Xu et al., 2019). We found that CTN increased the level of Nrf2, suggesting that CTN had anti-inflammatory effect on activated microglial cells. In addition, knocking down Nrf2 led to reversal of CTN’s inhibitory effect on LPS-induced production of NO, iNOS, COX2, and IL-1β, indicating that the anti-inflammatory effect of CTN is partially through the activation of Nrf2 in BV-2 microglial cells. One of the downstream molecules of Nrf2 is HO-1. The latter is a stress-induced antioxidative enzyme catalyzing the degradation of heme into carbon monoxide, biliverdin, and free ions in mammalian cells. Many studies have confirmed that HO-1 and metabolites from the enzymatic reaction have anti-inflammatory and neuroprotective effects (Li et al., 2014; Lv et al., 2018). For example, increased expression of HO-1 in dendritic cells can inhibit the secretion of pro-inflammatory cytokines induced by LPS (Chauveau et al., 2005). In the present study, CTN significantly increased the expression of HO-1 and knocking down HO-1 led to reversal of CTN’s inhibitory effect on LPS-induced production of iNOS and COX2. Therefore, it can be concluded that the anti-inflammatory effect of CTN is partially through the activation of HO-1.

We are the first to show that CTN increased the expression of Nrf2 and HO-1, which was regulated by the PI3K/Akt signaling pathway. Akt, also named protein kinase B, is one of the downstream molecules of PI3K. Under physiological and pathological conditions, the PI3K/Akt signaling pathway regulates cell proliferation, apoptosis, autophagy, and differentiation. The PI3K/Akt pathway also promotes cell survival by facilitating the translocation of Nrf2 to the nucleus (Lee et al., 2001; Nakaso et al., 2003). It also regulates the expression of HO-1 (Lee and Jeong, 2016). Our study found that CTN increased the level of Akt phosphorylation with time in BV-2 microglial cells. Inhibiting the PI3K/Akt pathway with LY294002 downregulated the expression of Nrf2 and HO-1 induced by CTN. These indicate that CTN induces activation of Nrf2 and HO-1 by activating the PI3K/Akt signaling pathway.

One of the interesting findings of our study is that downregulated expression of NLRP3 by CTN was related to the activation of Nrf2. This has not been reported by others. Inflammasomes are a type of cytosolic sensors detecting microbial infection, tissue injury, and metabolic imbalance. They stimulate the maturation and release of proinflammatory mediators including IL-1β and IL-18. A typical example is NLRP3 which plays a key role in the pathogenesis of multiple neurodegenerative diseases such as PD and AD (Zhou et al., 2016; Li et al., 2019). The present study is the first to report that CTN inhibits LPS-induced expression of NLRP3 and this is partially reversed when Nrf2 was knocked down in BV-2 microglial cells. It is, therefore, concluded that CTN inhibited LPS-induced NLRP3 expression partially through Nrf2.

## Conclusion

In conclusion, our *in vitro* results show that CTN has anti-inflammatory effect at concentrations non-toxic to cultured cells. This effect is mediated by activating NF-κB and the Nrf2/HO-1 signaling pathway. The latter is regulated by the PI3K/Akt signaling pathway. Due to the complexity of live tissues, the anti-inflammatory mechanism of CTN *in vivo* might be different from that of *in vitro* experiments. Future studies can test this anti-inflammatory mechanism of CTN *in vivo*. Our findings provide new insights into the anti-inflammatory mechanism of CTN and lay the theoretical foundation of preventing or attenuating neuroinflammation-related diseases, especially neurodegenerative diseases with activated microglial cells.

## Data Availability

The datasets generated for this study are available on request to the corresponding author.

## Author Contributions

YZ and PZ conceived and designed the study. DW and XW contributed to the data analysis. YZ drafted the manuscript. PZ, DW, and WY critically revised the manuscript. All authors read and approved the final manuscript.

## Conflict of Interest Statement

The authors declare that the research was conducted in the absence of any commercial or financial relationships that could be construed as a potential conflict of interest.

## Supplementary Material

FIGURE S1 Effect of CTN on the viability of BV-2 microglial cells. **(A)** Chemical structure of CTN. **(B)** BV-2 microglial cells were pretreated with CTN at various concentrations for 1 h and subsequently co-treated with 1 μg/ml LPS for 24 h. Cell viability was assessed using both intracellular and extracellular LDH assay. CTN at concentrations of 1, 5, and 10 μM did not influence the viability of cells, whereas CTN at 20 and 40 μM decreased the viability of cells. Data were presented as mean ± SEM. All experiments were repeated at least three times. ^∗^*P* < 0.05, ^∗∗^*P* < 0.01, ^∗∗∗^*P* < 0.001.

FIGURE S2 Effect of CTN on the morphology of LPS-activated BV2 microglial cells. BV2 microglial cells were pretreated with CTN at different concentrations for 1 h and then treated with LPS for 18 h. LPS significantly increased the area of microglial cells and their perimeters, whereas CTN can partially attenuate this increase. Data were presented as mean ± SEM. All experiments were repeated at least three times. ^∗^*P* < 0.05, ^∗∗∗^*P* < 0.001.
