# Supplementary figures and images for: Cryptotanshinone Attenuates Inflammatory Response of Microglial Cells via the Nrf2/HO-1 Pathway
Source: Front Neurosci. 2019 Aug 21;13:852. doi: 10.3389/fnins.2019.00852 (PMC6712928; doi:10.3389/fnins.2019.00852)

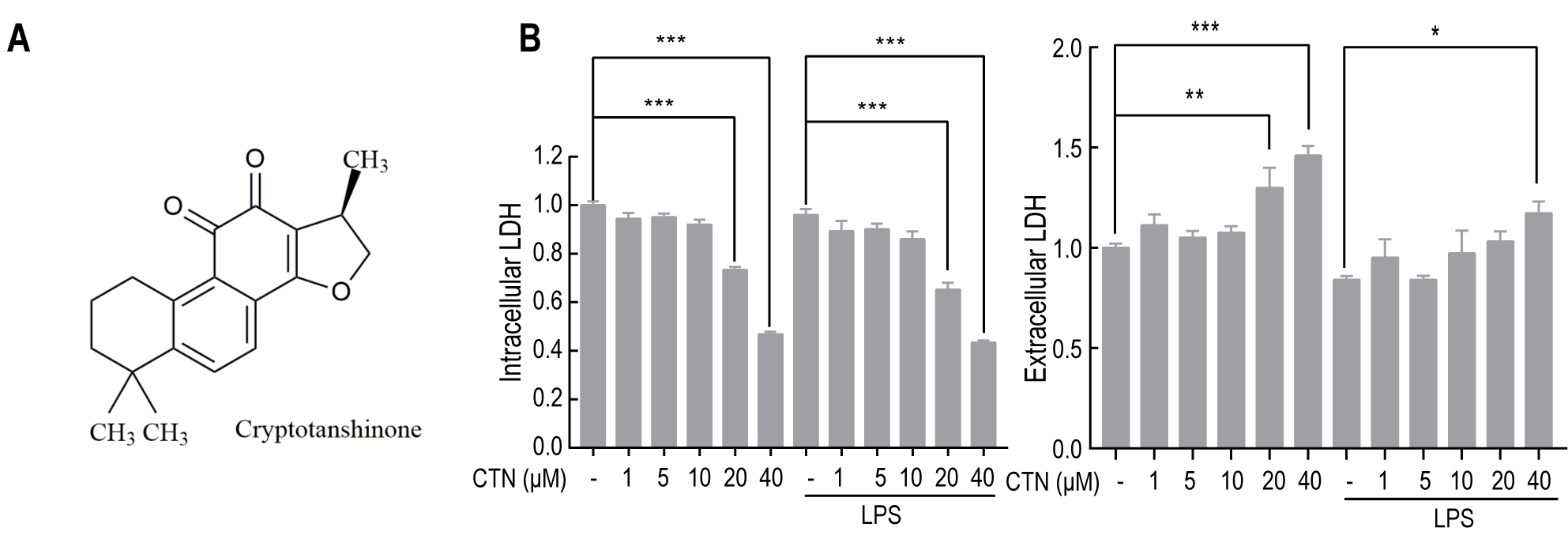

Supplement: FIGURE S1 — Effect of CTN on the viability of BV-2 microglial cells. (A) Chemical structure of CTN. (B) BV-2 microglial cells were pretreated with CTN at various concentrations for 1 h and subsequently co-treated with 1 μg/ml LPS for 24 h. Cell viability was assessed using both intracellular and extracellular LDH assay. CTN at concentrations of 1, 5, and 10 μM did not influence the viability of cells, whereas CTN at 20 and 40 μM decreased the viability of cells. Data were presented as mean ± SEM. All experiments were repeated at least three times. ∗P < 0.05, ∗∗P < 0.01, ∗∗∗P < 0.001. [file Image_1.tif]

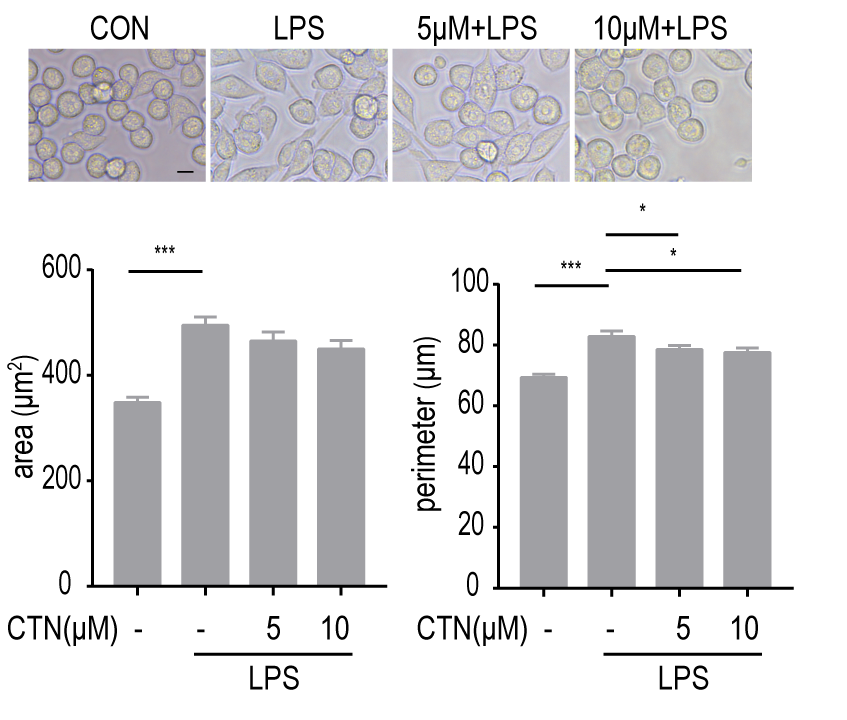

Supplement: FIGURE S2 — Effect of CTN on the morphology of LPS-activated BV2 microglial cells. BV2 microglial cells were pretreated with CTN at different concentrations for 1 h and then treated with LPS for 18 h. LPS significantly increased the area of microglial cells and their perimeters, whereas CTN can partially attenuate this increase. Data were presented as mean ± SEM. All experiments were repeated at least three times. ∗P < 0.05, ∗∗∗P < 0.001. [file Image_2.TIF]
